# Phylogeography of the Solanaceae-infecting Basidiomycota fungus *Rhizoctonia solani *AG-3 based on sequence analysis of two nuclear DNA loci

**DOI:** 10.1186/1471-2148-7-163

**Published:** 2007-09-13

**Authors:** Paulo C Ceresini, H David Shew, Timothy Y James, Rytas J Vilgalys, Marc A Cubeta

**Affiliations:** 1 ETH Zurich – Swiss Federal Institute of Technology, IBZ, Universitaetstrasse 2, LFW B28, 8092 Zurich, Switzerland/Universidade Estadual Paulista – UNESP, Departamento de Fitossanidade, Engenharia Rural e Solos, 15385-000, Ilha Solteira, São Paulo, Brazil; 2 Department of Plant Pathology, North Carolina State University, Raleigh, NC 27695, USA; 3 Department of Biology, Duke University, Durham, NC 27708, USA

## Abstract

**Background:**

The soil fungus *Rhizoctonia solani *anastomosis group 3 (AG-3) is an important pathogen of cultivated plants in the family Solanaceae. Isolates of *R. solani *AG-3 are taxonomically related based on the composition of cellular fatty acids, phylogenetic analysis of nuclear ribosomal DNA (rDNA) and beta-tubulin gene sequences, and somatic hyphal interactions. Despite the close genetic relationship among isolates of *R. solani *AG-3, field populations from potato and tobacco exhibit comparative differences in their disease biology, dispersal ecology, host specialization, genetic diversity and population structure. However, little information is available on how field populations of *R. solani *AG-3 on potato and tobacco are shaped by population genetic processes. In this study, two field populations of *R. solani *AG-3 from potato in North Carolina (NC) and the Northern USA; and two field populations from tobacco in NC and Southern Brazil were examined using sequence analysis of two cloned regions of nuclear DNA (pP42F and pP89).

**Results:**

Populations of *R. solani *AG-3 from potato were genetically diverse with a high frequency of heterozygosity, while limited or no genetic diversity was observed within the highly homozygous tobacco populations from NC and Brazil. Except for one isolate (TBR24), all NC and Brazilian isolates from tobacco shared the same alleles. No alleles were shared between potato and tobacco populations of *R. solani *AG-3, indicating no gene flow between them. To infer historical events that influenced current geographical patterns observed for populations of *R. solani *AG-3 from potato, we performed an analysis of molecular variance (AMOVA) and a nested clade analysis (NCA). Population differentiation was detected for locus pP89 (Φ_*ST *_= 0.257, significant at P < 0.05) but not for locus pP42F (Φ_*ST *_= 0.034, not significant). Results based on NCA of the pP89 locus suggest that historical restricted gene flow is a plausible explanation for the geographical association of clades. Coalescent-based simulations of genealogical relationships between populations of *R. solani *AG-3 from potato and tobacco were used to estimate the amount and directionality of historical migration patterns in time, and the ages of mutations of populations. Low rates of historical movement of genes were observed between the potato and tobacco populations of *R. solani *AG-3.

**Conclusion:**

The two sisters populations of the basidiomycete fungus *R. solani *AG-3 from potato and tobacco represent two genetically distinct and historically divergent lineages that have probably evolved within the range of their particular related Solanaceae hosts as sympatric species.

## Background

*Rhizoctonia solani *Kühn is a species complex composed of genetically distinct groups of fungi in the cantherelloid clade of the phylum Basidiomycota [1]. Current classification within the *R. solani *species complex is based largely on grouping of isolates into anastomosis groups (AG) based on their hyphal interactions. At least 13 AG have been described within the *R. solani *species complex including AG-1 to AG-13 [2-4]. Current knowledge suggests that AG and their subgroups represent independent evolutionary units within *R. solani *[5,6]. Anastomosis group 3 (AG-3) of *Rhizoctonia solani *Kühn is associated primarily with diseases of Solanaceous plants. Two well defined phylogenetic (sister) groups of *R. solani *AG-3 that cause diseases associated with potato and tobacco have been identified recently by analysis of sequence variation in ribosomal DNA (rDNA) [6-8] and beta-tubulin genes [9]: the AG-3 PT and TB. Isolates from the two hosts are closely related, but differ in their dispersal, epidemiology, fatty acid composition, and AFLP patterns [10-16]. Moreover, the host range of isolates of *R. solani *from potato and tobacco does not overlap, suggesting that they may represent genetically subdivided populations that have evolved a high level of specificity on different Solanaceous plant hosts [3,8,16,17].

The ecology and epidemiology of *R. solani *AG-3 on potato and tobacco has been extensively studied [3]. In general, isolates of *R. solani *AG-3 from potato are predominantly asexual and survive as mycelium and sclerotia in soil and on potato seed tubers [18]. When the sexual stage (teleomorph=*Thanatephorus cucumeris *Frank Donk) is formed during periods of cool and moist weather, meiospores (i.e., basidiospores) have limited dispersal and do not contribute directly to disease epidemics on potato [19]. However, sexual spores produced as a result of heterothallic mating, might constitute an important aspect of fungal life history that contributes to the genetic diversity and structure of field populations of *R. solani *AG-3 on potato. Ceresini et al [12] have recently suggested a model of population structure that includes both recombination and clonality for *R. solani *AG-3 on potato that provides experimental support for this assertion.

In contrast, isolates of *R. solani *AG-3 from tobacco are predominantly sexual and basidiospores of *T. cucumeris *serve as infectious propagules that initiate disease on tobacco leaves. However, the mating system of *T. cucumeris *from tobacco is not known. Likewise, it is not known whether recombination associated with sexual reproduction occurs among field isolates of *R. solani *AG-3 from tobacco [5,20].

Despite the evidence for genetic division among populations of *R. solani *AG-3 from potato and tobacco, many questions remain regarding their population biology and genetics. For example, although recombination had been detected within populations of *R. solani *AG-3 from potato, the role of asexual and sexual reproduction in determining population structure of *R. solani *AG-3 from potato is not known. Similarly, questions remain about the extent of gene flow among populations of *R. solani *AG-3 from tobacco and between populations of AG-3 from potato and tobacco.

For *R. solani *AG-3 from potato, a recent study supports the concept of migration of the pathogen on potato seed tubers from source populations from Northern US (Maine and Wisconsin) and Canada into NC, which provides evidence (based on classical *F *statistics) for a low level of genetic differentiation between source and recipient populations [21]. However, the causal role of contemporary gene flow on the observed population structure has not been determined. Traditional *F *statistics do not use temporal information on allelic variation, which would allow for inferences of evolutionary relationships. For example, there is no information revealing how current geographical patterns of genetic diversity in *R. solani *AG-3 from potato is influenced by population structure, history, and by a combination of structural and historical events.

In this study, four hypotheses were tested: 1) populations of *R. solani *AG-3 from potato and tobacco have different levels of genetic diversity; 2)populations of AG-3 from potato and tobacco differ in their extent of recombination; 3) there is no gene flow between populations of *R. solani *AG-3 from potato and tobacco; and 4) there is no significant association between haplotypes of AG-3 from potato and geographic location.

The tobacco population of *R. solani *AG-3 is hypothesized to have a higher level of genetic diversity and a predominant recombining structure due to the importance of sexual spores on disease epidemics. In contrast, populations of *R. solani *AG-3 from potato are hypothesized to have lower level of genetic diversity and a non-recombining structure, probably due to the predominant clonal reproduction system. The complete absence of gene flow between AG-3 from potato and tobacco is hypothesized as the reason for these populations being genetically unconnected. The hypothesis of no geographical association implies that populations of *R. solani *AG-3 from potato constitutes one single panmictic population with no genetic subdivision.

Therefore our goal was to analyze and further elucidate the phylogeography of populations of *R. solani *AG-3 from potato and tobacco. To address these phylogeographical hypotheses, we have focused on the observation, description and analysis of the spatial distribution of genotypes of *R. solani *AG-3 from potato and tobacco and the inference of historical scenarios based on coalescent gene genealogies [22,23]. In this study, we examined the sequence variation of two cloned nuclear DNA fragments from *R. solani *AG-3 from potato and tobacco. These regions of the genome were selected based on *a priori *information from seven PCR-RFLP markers previously used for genotyping *R. solani *AG-3 from potato and tobacco [12].

## Results

### Measures of nucleotide diversity and intragenic recombination

For the total population of 28 isolates of *R. solani *AG-3 sampled, 16 unique haplotypes were identified for locus pP42F and 22 for locus pP89 (Table 1). The haplotype (gene) diversity was 0.856 ± 0.048 and 0.869 ± 0.048 at pP42F and pP89, respectively. The average number of substitutions per site (π value) between two random samples was 0.03769 at pP42F and 0.03486 at pP89. The average number of nucleotide differences (k) was 15.113 [with a total variance V(k) of 5.403] at pP42F whereas at pP89 k was 37.862 (V(k) = 13.268). Of the total aligned 401 positions at pP42F, 45 (or 11.22%) were polymorphic. At pP89, from a total of 1086 positions, 98 (or 9.02%) were variable. Of the variable positions, 16 at pP42F (or 35.56%) and 22 at pP89 (or 22.45%) were unique to a single sample.

**Table 1:** Descriptive analysis of molecular variation within two cloned nuclear DNA fragments from samples of *Rhizoctonia solani *AG-3 isolates from potato and tobacco

Sample of isolates	Geographical origin	Total number of mutations, Eta	Number of polymorphic (segregating) sites	Number of haplotypes, NHap	Haplotype (gene) diversity ± standard deviation	Nucleotide diversity, Pi	Average number of nucleotide differences, k	Sampling variance of k, Vs(k) ^b^	Total variance of k, V(k) ^b^
**Locus^a^**	**pP42F^a^**								
Total sample: potato and tobacco		48	45	16	0.856 ± 0.048	0.03769	15.113	0.265	5.303
Potato	Eastern NC, Maine and Wisconsin	22	20	14	0.942 ± 0.026	0.01340	5.373	0.156	1.947
Tobacco	Central NC and Southern Brazil	11	11	2^b^	0.133 ± 0.112	0.00366	1.467	0.070	0.559

**Locus^a^**	**pP89^a^**								
Total Sample: potato and tobacco		100	98	22	0.869 ± 0.048	0.03486	37.862	0.647	13.268
Potato	Eastern NC, Maine and Wisconsin	32	32	20	0.963 ± 0.029	0.00703	7.640	0.212	2.759
Tobacco	Central NC and Southern Brazil	16	16	2^b^	0.133 ± 0.112	0.00196	2.133	0.102	0.813

^a ^The total number of sites analyzed (excluding sites with alignment gaps) was 401 (pP42F) and 1090 (pP89).

^b ^The variances of k were estimated assuming free recombination.

^c ^One single allele was detected in the North Carolina sample of tobacco isolates.

For the potato sample of *R. solani *AG-3, 14 unique haplotypes were identified for locus pP42F and 20 for locus pP89 (Table 1); whereas, for AG-3 from tobacco only two haplotypes were identified at both pP42F and pP89. Consequently, higher haplotype diversity was observed for AG-3 from potato (0.942 ± 0.026 at pP42F and 0.963 ± 0.029 at pP89). In contrast, for AG-3 from tobacco, a considerably smaller value of haplotype diversity (0.133 ± 0.112) was observed at both pP42F and pP89. The average π value between two random samples was 0.01340 at pP42F and 0.00706 at pP89 for AG-3 from potato, whereas for *R. solani *AG-3 from tobacco smaller π values ranging from 0.00196 (pP89) to 0.00366 (pP42F) were observed. For *R. solani *AG-3 from potato, the average number of nucleotide differences (k) was 5.373 [V(k) = 1.947] at pP42F and 7.64 [V(k) = 2.759] at pP89. The average k values for AG-3 from tobacco were smaller and ranged from 1.467 [V(k) = 0.559] at pP42F to 2.133 [V(k) = 0.813] at pP89. Of the total aligned 401 positions at pP42F, 20 (or 4.99%) were polymorphic for AG-3 from potato and 11 (or 2.74%) for AG-3 from tobacco. At pP89, from a total of 1086 positions, 32 (or 2.95%) were variable for *R. solani *AG-3 from potato and 16 (or 1.47%) for AG-3 from tobacco.

**Figure 1:**
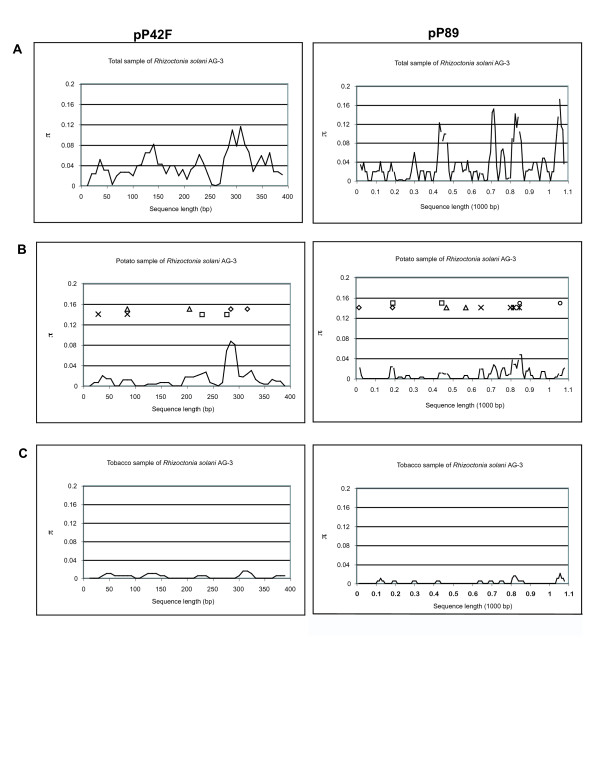
**Spectrum of polymorphism among the two cloned nuclear loci pP42F and pP89 depicted as sliding window of size 25 bp and step size of 8 bp**. π describes the average number of pairwise substitutions at a particular site among the total population (A), the potato (B) and the tobacco (C) populations of *Rhizoctonia solani *AG-3. The abscissa represents the position along the spacer region about which the sliding window is centered. Geometric figures of similar shape inside section B of the figures represent within-locus recombination sites.

The spectrum of polymorphism along each locus (which was measured as π along a sliding window of 25 positions; Figure 1) contrasts the high nucleotide diversity of the combined total sample of *R. solani *AG-3 with the potato and tobacco samples separately. Most of the nucleotide diversity was observed between samples of potato and tobacco *R. solani *AG-3. Within-sample comparison indicated higher nucleotide diversity for *R. solani *AG-3 from potato than for *R. solani *AG-3 from tobacco. For AG-3 from potato, recombination sites along the loci are indicated by geometric figures within the sliding windows. A minimum of four recombination events (Rm, [24]) was detected at pP42F: between sites 29 and 84, 84 and 203, 230 and 280, 285 and 319. The recombination parameter, R [25], was estimated as 19.3 per gene and 0.0482 between adjacent sites. At pP89, a minimum of six recombination events were detected: between sites 16 and 190, 190 and 444, 469 and 569, 647 and 800, 816 and 845, 845 and 1055. The estimate of R was 30.2 per gene resulting in 0.0277 between adjacent sites. In contrast, no recombination events were detected for AG-3 from tobacco. The estimate of R, per gene, was 0.001 and 0.000 between adjacent sites at both pP42F and pP89.

### Phylogenetic analysis

**Figure 2:**
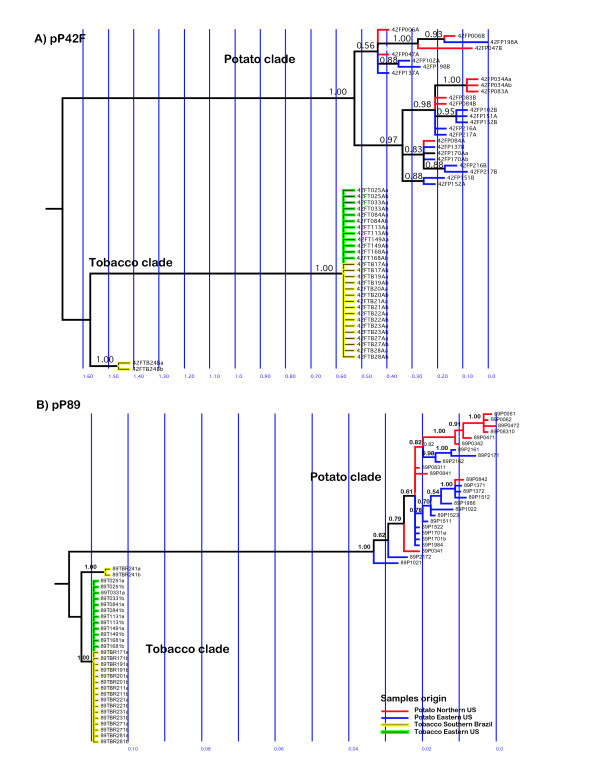
**Maximum likelihood (ML) trees of *Rhizoctonia solani *AG-3 based on sequence variation on two cloned nuclear DNA fragments: A) pP42F and B) pP89. The trees are based on the evolutionary model K80 (K2P) + G (for pP42F) and +G+I (for pP89) for DNA bases substitution**. Values on the x-axis indicate expected changes per site. Values above particular tree partitions (varying from 0 to 1.0) are posterior probability credibility values for the clades generated by Bayesian MCMCMC analyses. For example, a credibility value of 0.99 indicates the proportion of trees sampled (a total of 10892 among 11002 total trees), which contains the partition for the respective clade.

Two major clades in *R. solani *AG-3 were inferred from the Bayesian phylogenetics analysis of the sequence variation at the two cloned nuclear DNA fragments pP42F and pP89 (Figure 2-A and 2B): the potato and tobacco clades. There was strong support (credibility value = 1.00) for the splitting of the potato and tobacco groups of *R. solani *AG-3 haplotypes (represented by 16 or 56 changes in the pP42F and pP89 loci, respectively). There were few other well-supported branches or clades (by high Bayesian credibility values) indicated in the phylogenetic gene tree. For pP89 (e.g.), one of these well-supported clades included only haplotypes from Northern US (89P0061, 89P0062, 89P0472, 89P08310, 89P0471, and 89P0342). There was strong support for a bifurcating branch that split the tobacco isolates into two distinct groups; one of which contained a haplotype detected in Brazil.

### Intraspecific evolution of *R. solani *AG-3 inferred by analysis of haplotype networks

**Figure 3:**
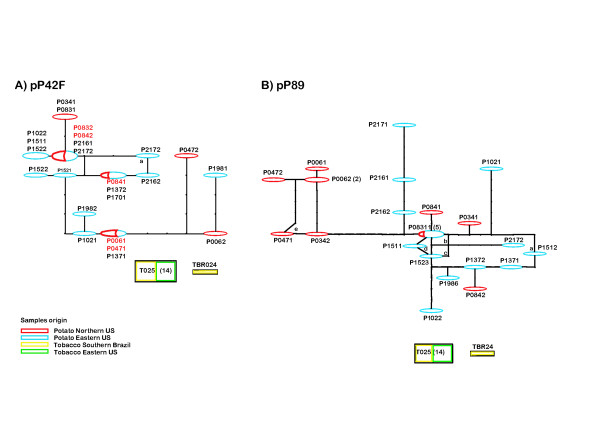
**Network of haplotypes of locus pP42F (A) and pP89 (B) of *Rhizoctonia solani *AG-3 constructed using the statistical parsimony algorithm [59] implemented by TCS [61]**. Geometric figures group haplotypes and the area of each figure represents the relative frequency of haplotypes in the population. Dots without denomination along the network indicate putative haplotypes not sampled from the population. Letters (a, b, c, d, and e) indicate probable homoplasy. The AG-3 TB haplotypes T025 and TBR24 were kept unconnected to the nested network because of the large mutational distance (a maximum of eight steps for pP42F and 14 for pP89) that separates them from the AG-3 PT haplotype by statistical parsimony analysis.

The reconstruction of haplotypes network of both pP42F and pP89 loci by statistical parsimony (Figure 3-A and 3B) supported the hypothesis of divergent evolution of genes from *R. solani *AG-3 from potato and tobacco. The degree of evolutionary relatedness was represented by mutational connections. There was no mutational connection linking any of the haplotypes of *R. solani *AG-3 from potato with haplotypes from tobacco in this network. The number of mutations splitting these two groups exceeded the calculated maximum connection steps (at 95%) of eight for pP42F haplotypes and 14 for pP89. The two haplotypes identified in the tobacco sample were also unconnected. This method also allows for the identification of the putative ancestral haplotype based on its frequency in the population. The most frequent allele is, theoretically, the oldest and could be identified, as the most interior to the network, showing several mutational connections [26]. However, the high gene diversity within *R. solani *AG-3 from potato did not allow for precise identification of the ancestral haplotype. Within-sample analysis of *R. solani *AG-3 from potato identified probable recombinants through observations of sequence homoplasy in the networks of both pP42F (point a) and pP89 loci (points a, b, c, d, and e).

### Population structure

Analysis of the population structure using AMOVA was conducted independently for both pP42F and pP89 loci. By grouping molecular sequencing haplotypes of *R. solani *based on their host of origin (either from potato or tobacco), most of the molecular variation was detected between potato and tobacco populations (87.7 to 92.0% of the total variance), with very little variation among geographical populations within each group (0.4 to 1.7%). The within-population variance corresponded to 11.9 and 6.3% of the total variance for pP42F and pP89 respectively. The overall Φ_ST _was 0.88 for pP42F and 0.94 for pP89 (P ≤ 0.001).

Pairwise comparison between geographic populations was performed using estimates of F_ST _equivalents (pairwise Φ_ST _values) for both pP42F and pP89 loci separately (Table 2). Pairwise Φ_ST _values were significantly greater than zero, which would indicate population differentiation, mainly for comparisons between potato and tobacco populations of *R. solani*. For example, the potato populations from Northern US or Eastern NC were significantly different from both tobacco populations from Central NC or Southern Brazil. There was no indication of differentiation between the tobacco populations of Central NC and Southern Brazil. In contrast, while no subdivision or differentiation was observed between the potato populations from Northern US and Eastern NC for the pP42F locus (Φ_*ST *_= 0.034, not significant), differentiation between these two populations was observed for the pP89 locus (Φ_*ST *_= 0.257, significant at P < 0.05).

**Table 2:** Population pairwise Φ_ST _^a ^of *Rhizoctonia solani *AG-3 PT and TB collected from commercial potato or tobacco fields in Northern US, Eastern and Central North Carolina and Southern Brazil for loci pP42F (above diagonal) and pP89 (below diagonal)

Locus					pP42F					
	Source		Potato		Potato		Tobacco		Tobacco	
	
		Origin	Northern US		Eastern NC		Central NC		Southern Brazil (PR, SC)	
	
	Potato	Northern US	-		0.03414	^NS^	0.9017	*	0.8656	*
	Potato	Eastern NC	0.2567	*	-		0.8968	*	0.8682	*
pP89										
	Tobacco	Central NC	0.9466	*	0.9460	*	-		0.0270	^NS^
	Tobacco	Southern Brazil (PR, SC)	0.9285	*	0.9313	*	0.0270	^NS^	-	

^a ^Population pairwise Φ_ST _was calculated using ARLEQUIN version 2.000 [64]. Asterisks indicate significance of *P *values at *P *≤ 0.05 (*) leading to values of Φ_ST _larger than or equal to the observed value when permuting molecular sequencing genotypes between populations (1000 permutations were performed).

### Nested clade analysis

**Figure 4:**
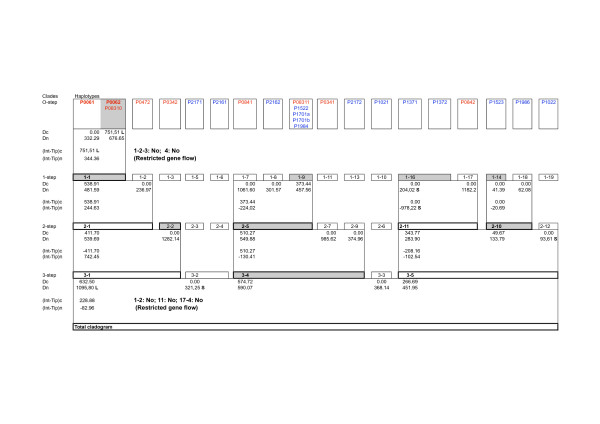
**Results of the nested cladistic analysis of geographical distance for pP89 haplotypes of *Rhizoctonia solani *AG-3 from potato**. The haphotype designations are given at the top and are boxed together to reflect one-step nested design. Higher-level clade designations are given as moving down the figure, with boxed grouping indicating the nesting structure. Immediately below each clade designation is the clade (D_c_) and nested clade (D_n_) distances respectively. An **S **indicates the distance is significantly small at the 5% level, and an **L **indicates that it is significantly large. For nested clades in which the tip/interior status is known and for which both tips and interior exists within the same nesting groups, the clade name and distances are shaded for interior clades and are left unshaded for tip clades. At the bottom of the boxes that indicate the nested groups containing both tip and interior clades the lines indicated by (Int - Tip)_c _and (Int - Tip)_n _give the average difference in distances between the interior clades and tip clades within the nested group for clade distances and nested clade distances, respectively. NA stands for non-applicable. The chain of inference for clades with significant historical pattern (e.g. clades 1-1 and total cladogram for pP89 haplotypes) follows the key provided in the Appendix of Templeton [34].

To discriminate between phylogenetic patterns resulting from historical events from those due to recurrent gene flow, we performed nested clade analysis on the haplotype network of *R. solani *AG-3 from potato obtained by statistical parsimony analysis and presented in Figure 3-B. The resulting nesting design and the NCA are presented in Figure 4. No NCA was performed for the pP42F haplotype network (Figure 3-A) due to no population subdivision observed between Eastern NC and Northern US for this locus.

For the NCA of pP89, high sequence variation was observed, and such variation has caused a few cases of ambiguity (probably due to recombination) in the network estimation. As the NCA does not accommodate recombination, haplotypes P0471, P1511 and P1512 were removed from the reticulate network presented in Figure 3-B. A significant geographical association pattern indicating restricted gene flow with isolation by distance was found (Figure 4). The interior haplotypes P0062 and P08310 from Northern America contained in the one-step clade 1-1 showed significantly large (P < 0.05) *D*_*c*_. In addition, these internal haplotypes showed larger *D*_*c *_than the average clade distance for the tip haplotype P0061 that has presumably descended from it [i.e. (*Int *- *Tip*)_*c *_significantly large at P < 0.05]. At the higher level, within the entire cladogram nesting, the tip clade 3-2 (that included the two-step level clades 2–3 and 2–4, containing only haplotypes from Camden, NC), showed significantly small *D*_*c*_, indicating restricted gene flow as well. In contrast, clade 3-1 showed a significantly large *D*_*c*_, which would indicate a long distance dispersal event, probably from Wisconsin to Maine, which contained the tip haplotypes. Clade 3-1 includes the two-step clades 2-1 (composed by one-step clades 1-1 and 1–2) and 2-2 (which contained the one-step clade 1–3). This 3-step clade contained haplotypes only observed in Northern America.

### Coalescent analysis

Neutrality tests were performed for each locus and populations of *R. solani *AG-3, indicating neutral evolution of both loci for the populations from potato. Evidence of non-neutral evolution was found in both pP42F and pP89 loci for the pooled populations of AG-3 from tobacco [Table 3]. For these two loci in the tobacco (TB) group the significant statistical values were negative. A significant test result is consistent with either population growth or shrinkage, or background selection [27]. We hypothesized that the deviation from neutrality observed for the TB group was due to a decline in effective population size. This hypothesis was supported by the exponential population growth estimates (*g*) [28,29]. The *g *values for both pP42F and pP89 were negative for the TB group, indicating population shrinkage [Table 4]. In contrast, positive *g *values indicated that the populations from potato were growing. However, *g *values are not symmetrical in magnitude due to its exponential effect on the population growth [Theta_t _= Theta_presenttime _exp(-gt), t = a time before present]. Thus, a *g *= 10 would indicates a rather slow growth while a *g *= -10 indicates a significant shrinkage of the population.

**Table 3:** Estimates from neutrality tests for each locus and populations of *Rhizoctonia solani *AG-3 from potato and tobacco

Loci and populations		Fu and Li's D		Fu and Li's F*		Tajima's D	
pP42F							
1.	Potato (Northern US + Eastern NC)	-0.580	^NS^	-0.516	^NS^	-0.569	^NS^
2.	Tobacco (US + Brazil)	-4.401	*	-3.662	*	-2.228	*
pP89							
1.	Potato (Northern US)	0.352	^NS^	0.503	^NS^	0.740	^NS^
2.	Potato (Eastern NC)	0.399	^NS^	0.453	^NS^	0.467	^NS^
3.	Tobacco (US + Brazil)	-4.798	*	-3.905	*	-2.337	*

^NS ^Non significant or * significant at *P *≤ 0.05

**Table 4:** Estimates of coalescent parameters from the divergence between potato and tobacco populations of *Rhizoctonia solani *AG-3

Loci and populations		Effective sample size	Population growth rate ^a^	Theta (4N_e _μ) ^b^	Migration rate ^c ^4Nm (+ = receiving population)		
pP42F					1, +		2, +
1.	Potato (Northern US + Eastern NC)	26	37.05	0.01412	-		0.0000
2.	Tobacco (US + Brazil)	30	-23.63	0.00422	0.0994		-
pP89					1, +	2, +	3, +
1.	Potato (Northern US)	10	34.12	0.00203	-	5.1115	0.0000
2.	Potato (Eastern NC)	16	10.21	0.00155	0.1258	-	0.0417
3.	Tobacco (US + Brazil)	30	-36.95	0.00176	0.0000	0.0000	-

^a ^Most probable estimate of population growth rate calculated by Bayesian analyses using the program Lamarc 2.0 [28, 29].

^b ^Theta values represent a measure of effective population size (for diploids, Theta = 4Neμ, where Ne = effective population size and μ = mutation rate inferred for each locus).

^c ^Migration between geographical or host populations was estimated using an isolation with migration model in the program Migrate implemented by SNAP Workbench [68]. Source populations are shown along the top and sink populations are indicated on the left. Estimates of population's growth, theta and migration rates are at 95% confidence interval.

The population parameter Theta was used as a relative measure of effective population size. The estimates of Theta are summarized in Table 4. The pattern observed for the pP42F locus suggests a larger effective population size for the pooled *R. solani *AG-3 populations (Northern US + Eastern NC) from potato (Theta = 0.01412) when compared to the pooled AG-3 populations (US + Brazil) from tobacco (Theta = 0.00422). However, for the pP89 locus the Theta values ranged from 0.00155 to 0.00206 for the populations of AG-3 from potato from Eastern NC and Northern US, respectively, and 0.00176 for the pooled population samples of *R. solani *AG-3 from tobacco.

Estimates of directional gene flow between populations of *R. solani *AG-3 from potato and tobacco were consistent with relatively low rates of migration between them and ranged from 0 to 0.0994. When the pooled populations of *R. solani *AG-3 from potato were compared with the pooled populations from tobacco for the pP42F locus, the estimates of directional gene flow (since the divergence of *R. solani *AG-3) were very low, with a historical movement of genes from the pooled potato populations (source) to the pooled tobacco populations (recipient) of *R. solani *AG-3 (4Nm_1→2 _= 0.0994). For the pP89 locus, there was no evidence of migration between either of the two populations from potato to the pooled populations from tobacco. However, the migration parameter was estimated to be 0.0417 (4Nm_3→2_) from the pooled tobacco populations to the Eastern NC potato population. While the highest historical migration contribution was observed from the Eastern NC to the Northern US potato population of AG-3 (migration rate 4Nm_2→1 _= 5.1115), a much smaller migration value was observed in the opposite direction (4Nm_1→2 _= 0.1258).

**Figure 5:**
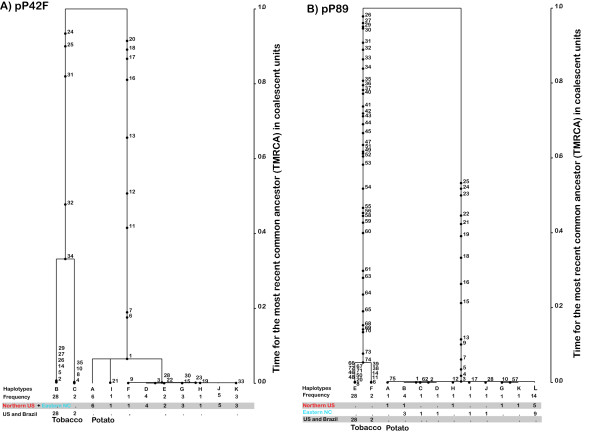
**Coalescent-based gene genealogies with the highest root probability [L = 1.3438e-53, SD = 1.3225e-53 for A) pP42F, and L = 2.5718e-96, SD = 1.8275e-96 for B) pP89 locus] showing the distribution of mutations in the two major clades of *Rhizoctonia solani *AG-3 (the AG-3 PT from potato ad AG-3 TB from tobacco)**. Mutations and bifurcations are time ordered from the top (past) to the bottom (present). The mutations designations are correspondent to respective site numbers described in the additional files 1 and 2. The inferred gene genealogies are based on 100,000 simulations of the coalescent with Waterston's estimates of Θ (M) = 2.57 for pP42F and 2.27 for pP89. The time scale is expressed in coalescent units of 4N (where N is the populations size). The letters below the tree designate the distinct haplotypes, their total or geographically distinct observed frequencies. Based on estimates of gene flow between the Northern US and Eastern NC populations from AG-3 PT (Table 2), the coalescent was inferred pooling the two populations of the pathogen for the pP42F locus.

From the coalescent analyses, the overall tree topologies and the relative divergence of *R. solani *AG-3 from potato and tobacco were consistent for both pP42F and pP89 loci. The ancestral distribution of mutation and coalescent events for the two loci are presented in Figure 5. The branching between the populations of *R. solani *AG-3 from potato and tobacco occurred at the deepest point, suggesting an ancient divergence between them. The tobacco populations of AG-3 represent the oldest lineages within the trees (when compared with the populations from potato), and estimates of divergence are five times greater in magnitude. The relative ages of the populations from tobacco varied from 0.05 (for the pP89 locus) to 0.34 units of coalescent time (for the pP42F locus). Populations from potato have experienced a recent expansion as demonstrated by the diversification of haplotypes in both gene trees and were inferred to be younger than those from tobacco.

## Discussion

We have studied two closely related sister groups within the basidiomycete fungus *R. solani *AG-3 on taxonomically related hosts and this system provided us with a unique opportunity to examine aspects of their population genetics in a phylogeographic framework.

During the process of screening of markers for population genetics, four of seven PCR-RFLP markers previously used for genotyping isolates of *R. solani *AG-3 from potato (pP42, pP46, pP47 and pP89) also amplified genomic DNA from *R. solani *AG-3 from tobacco. Except for marker pP42F, none of the alleles present in the sample of 20 tobacco isolates of *R. solani *AG-3 from tobacco were shared with the potato sample [12]. As these markers were shown to be conserved enough to amplify genes across potato and tobacco populations of AG-3, we explored their potential appropriateness for phylogenetic and population genetics analyses within *R. solani *AG-3. For this study, we selected two (pP42 and pP89) of these four genetic markers. An important outcome from the sequencing analysis of these two markers (pP42 mad pP89) was the increasing power for discriminating alleles within a locus. Genotyping by PCR-RFLPs indicated the occurrence of only two or three alleles (for pP42F and pP89 loci, respectively) within a potato population of *R. solani *AG-3 from NC [12]. However, genotyping by sequencing analysis indicated that the actual number of alleles present within that population was much higher, with 14 at pP42F and 20 at pP89.

The first objective of this study was to determine the extent of genetic divergence between populations of *R. solani *AG-3 from potato and tobacco. The potato sample was more genetically diverse showing a higher number of nucleotide differences among haplotypes, which resulted in high haplotype (gene) diversity. In contrast with the sample from potato, limited to no genetic diversity was observed within the tobacco sample from NC and Brazil. The number of nucleotide differences was considerably smaller for tobacco than for potato isolates of *R. solani *AG-3. Only two haplotypes were found for the tobacco population in Brazil, with a single haplotype predominating in North America. Our results support the first hypothesis that populations of *R. solani *AG-3 from potato and tobacco have different levels of molecular diversity. Consistent with the heterokaryotic nature of *R. solani*, every isolate of AG-3 from potato had at least one heterozygous locus. However, all tobacco isolates from both NC and Brazil were homozygous.

The other specific objective of this study was to determine how populations of *R. solani *AG-3 from potato and tobacco are shaped by recombination. We addressed this objective by analyzing the intragenic recombination at pP42 and pP89 loci. This analysis was done by determining both the recombination parameter R within a locus [25] and the minimum number of recombination events between two adjacent sites at the same locus [24].

The tobacco population of *R. solani *AG-3 was hypothesized to have a predominantly recombining structure due to the importance of sexual spores in disease epidemics. In theory, progeny generated via this type of reproduction would be genetically different than parental individuals as a result of recombination of genetically different nuclei [5,30-32]. In contrast, the potato population of AG-3 was hypothesized to have a non-recombining structure, probably because of the predominant clonal reproduction system.

Higher values of R were detected for the population of *R. solani *AG-3 from potato. In addition, a minimum of four recombination events were detected between sites along the pP42 locus and six recombination events at pP89 for the isolates of AG-3 from potato. Putative recombinants were also identified through observations of homoplasy in the networks of both pP42F and pP89 loci (Fig.4. 4A–B). In contrast, no recombination events were detected for AG-3 isolates from tobacco. Our data support the alternative hypotheses of a sexual recombining structure for *R. solani *AG-3 from potato. This is in agreement with the common occurrence and observation of the sexual stage in potato fields and previous research results that suggested recombination was occurring in population of AG-3 from potato based on analyses of the population structure of the fungus with seven co-dominant PCR-RFLP markers [12]. All seven loci were found in Hardy Weinberg Equilibrium and no linkage disequilibrium was detected for any pair of loci [12].

In contrast with the potato population, we found no evidence of recombination for the tobacco population. Fungal progeny generated via asexual or homothallic sexual (self-fertile, inbreeding) reproduction would be nearly genetically identical to the parental individuals (and all parts of the genome will have similar evolutionary history) in the absence of outcrossing. No or limited outcrossing would contribute to the association between independent characters (genetic markers), a population with limited genetic diversity and the repeated recovery of similar genotypes (e.g. clonal population structure) and new genotype in the population would arise mainly as a result of mutation [32].

Our previous observation indicated a clonal population structure for AG-3 from tobacco in NC based on the one-to-one (strict) association between two independent criteria (somatic incompatibility and AFLP markers) [11]. However, our current data is not sufficient for supporting the alternative hypothesis of clonality for AG-3 from tobacco because only two haplotypes were detected for each locus in the tobacco populations. The limited amount of genetic variation in this sample would not be sufficient to accurately assess the relative contribution of recombination in the pathogen population.

The third hypothesis tested in this study was that no gene flow occurs between populations of *R. solani *AG-3 from potato and tobacco, indicating that they constitute genetically distinct groups. The objective was to describe this pair of populations of *R. solani *AG-3 by the proportion of loci that show unilateral or reciprocal fixation, and by standard estimates of statistics based on gene flow.

In recently evolved species pairs it is expected to identify variable nucleotide positions where polymorphisms are shared by both sibling species, other nucleotide positions where only one species is polymorphic and the other is fixed for one allele, and where both species have fixed loci [11,33]. An analysis of the DNA polymorphisms at each locus, suggested that most of the nucleotide diversity occurred between samples of potato and tobacco *R. solani *AG-3. As genetic isolation is considered to precede the loss of shared polymorphism, discovering a single locus or multiple loci that show fixation in one or the other of the phylogenetic species would be evidence of genetic isolation [11,33]. No alleles were shared between the potato and tobacco populations of *R. solani *AG-3 used in this study. In addition, analysis of population structure indicated no gene flow between populations of *R. solani *AG-3 from potato and tobacco. Most of the molecular variation and differentiation was detected between potato and tobacco populations of *R. solani *AG-3. In contrast, a high level of gene flow was detected for tobacco group of *R. solani *AG-3. With only one exception (isolate TBR24), all of the isolates from NC and the Brazilian population of AG-3 from tobacco shared the same alleles at both pP42 and pP89 loci.

From the Bayesian phylogenetics analysis of cloned nuclear DNA fragments pP42F and pP89, two major clades in *R. solani *AG-3 were inferred, splitting haplotypes from potato and tobacco into two groups. In addition, the reconstruction of the haplotype network of both pP42F and pP89 loci by statistical parsimony indicated no mutational connection linking any of the haplotypes of *R. solani *AG-3 from potato and tobacco in this network. These results support the hypothesis of divergent evolution of genes from populations of *R. solani *AG-3 from potato and tobacco.

The fourth hypothesis tested was that there is no significant association between haplotypes of AG-3 from potato and geographic origin, which implies that there is a single panmictic population with no genetic subdivision.

Based on *F *statistics, an important observation about gene flow was inferred from the within-group analysis of population subdivision of the sample of *R. solani *AG-3 from potato. While gene flow (Φ_*ST *_= 0.03414, not significant) was detected between populations of *R. solani *AG-3 from potato at the pP42F locus, population differentiation was detected for the locus pP89.

To infer historical events influencing the current geographical patterns observed on populations of the pathogen, we performed NCA analysis on a reticulated phylogeny of AG-3 haplotypes from potato. The main benefit of using haplotype tree information is its qualitative advantage of discriminating among various biological explanations for any detected geographical association. By using a haplotype network analysis it was possible to examine spatial and temporal patterns of genetic variation in populations of *R. solani *AG-3 from potato whereas the *F*-statistics analysis could only examine the current spatial pattern.

Based on NCA, the null hypothesis of no association between haplotype variation and geography was rejected for pP89 locus. For this locus, the first prediction for the population structure hypothesis was that restricted gene flow caused the geographical associations. Within clade 1-1 from Northern America, clade distance equal to zero was associated with the tip pP89 haplotype P0061 whereas significantly large clade distance was associated with the interior haplotypes P0062 and P08310. In addition, the average interior clade distance minus the average tip clade distance was significantly large. These observations fulfilled the prediction that under restricted gene flow younger or tip clades should be less widespread relatively to older clades interior to them [34,35]. This is because restricted gene flow implies only limited movement by individuals during any giving generation as it takes time for a new haplotype to spread geographically. Another aspect of restricted gene flow is that the new haplotypes initially reside within the range of its ancestor for many generations, which is predicted by the isolation by distance model. In contrast, the ancestor would have a wider geographic distribution [36].

At the higher clade level the tip clade 3-2 (that included the two-step level clades 2–3 and 2–4, containing only haplotypes from Camden, NC), showed significantly small *D*_*n*_, indicating restricted gene flow. In contrast, clade 3-1 showed a significantly large *D*_*n*_, which would indicate a long distance dispersal event, probably from Wisconsin to Maine, which contained the tip haplotypes. However, conclusions concerning a long distance dispersal event should be interpreted cautiously since our sampling procedure was geographically limited. Therefore, it may not be possible to discriminate between isolation by distance and long distance gene flow models [35].

The patterns of spatial structure and differentiation between potato populations of *R. solani *AG-3 revealed by the two distinct markers were conflicting and deserve further discussion. Discordant patterns of divergence in molecular markers and loci are commonly interpreted as differences in mutation rate, neutrality or linkage disequilibrium with other loci that are subject to selection, and the differential effect of natural selection on those markers and loci [37-40]. Although we do not have ancillary information on whether the genetic loci examined in this study have distinct mutation rates, our data suggest a model of neutral evolution for both loci (Table 3). A plausible explanation for the conflicting patterns of spatial structure is due to the distinct resolution capabilities associated with difference in per-locus sequence length. For pP42 we detected 20 polymorphic sites in 401 bp and for pP89 we detected 32 segregating sites in 1090 bp. The paucity of informative sites on pP42F resulting from the analyses of less sequence length might have introduced a downward bias in the estimate of population differentiation. Support for this assertion is based on simulation data on how many loci are needed and how much sequence is required from each locus to provide better estimates of population genetic parameters [41].

In this study, we also conducted coalescent-based simulations of genealogical relationships between populations of *R. solani *AG-3 from potato and tobacco to estimate the amount and directionality of historical migration patterns in time, the ages of mutations and of populations (Table 4; Figure 5). This is the first time that a genealogical approach (based on the coalescent model) was used to study the population genetics and evolution of a *Rhizoctonia *pathosystem.

Tests of non-neutral evolution could not be rejected for the two loci, considering only the populations from tobacco. We believe that the observed patterns of gene diversity is most likely due to changes in population size and not to a intrinsic feature of the loci. Given the contemporary Theta values [42] it was possible to extrapolate the relative increase or decrease in population size since the time of divergence. Growth rate estimates [28] were consistent with a significant decrease in size for the tobacco populations of *R. solani *AG-3. We hypothesize that this decrease in size reflects a split where only a small fraction of the ancestral pathogen population founded a new population during the domestication of tobacco from the wild habitats into agricultural fields. In contrast, results from Lamarc analyses [28] were consistent with expansion of the potato populations of *R. solani *AG-3. The increase in *R. solani *AG-3 effective population size on potato was most likely caused by a corresponding increase in host population size following the expansion of agriculture.

Although characterized as being younger than the tobacco population of AG-3, the potato population has experienced a very recent diversification in the US. In contrast, the two tobacco populations sampled from Southern Brazil and Central NC (US), had a small effective population size. Older ancestor populations with higher haplotype diversity of *R. solani *(and consequently higher effective population sizes) might exist in the center of origin of tobacco and other related Solanaceae.

Our findings corroborate previous information that populations of *R. solani *AG-3 are subdivided by host specialization, constituting two distinct phylotypes [3,7,8,11,16,17]. *R. solani *AG-3 represents at least two phylogenetic species that display high phenotypic differences in disease biology and ecology [3,16,17].

Low rates of historical movement of genes were observed between the potato and tobacco populations of AG-3. These two populations of *R. solani *AG-3 represented two distinctly and historically divergent lineages, which have probably evolved within the range of their particular hosts as sympatric species. Reinforcement is considered as an important mechanism of speciation between sympatrically divergent populations [43]. Reinforcement is the active mechanism of reproductive isolation due to reduced fitness of the interspecific hybrids, thereby favoring intraspecific mating and resulting in genetic differentiation between species [44]. Reproductive success between *R. solani *AG-3 from potato and tobacco was not tested in our study, but this testing could provide knowledge about the mechanisms involved in sympatric speciation of *R. solani *AG-3.

The contrasting ecological context of these two host specific populations makes our study a good example of ecological speciation between lineages of plant pathogenic fungal populations. Host range and disease causing ability are clearly important factors in fungal speciation. Consistent with our study, evidence for lineage divergence strongly associated with closely-related host species has been previously reported for other fungi, such as *Magnaporthe grisea *and *Mycosphaerella graminicola *[43,45,46]. Several studies have also reported extensive genetic differentiation between divergent fungal species [47-51].

The results presented in this paper provide important insight into our understanding of the phylogeography of populations of *R. solani *AG-3 from potato and tobacco. The next step of our research will involve a more in depth study to reveal the origins and patterns of domestication of these two phylogenetically distinct populations of *R. solani *AG-3. Our experimental approach will involve a global sampling strategy for isolation of *R. solani *AG-3 from *Solanum tuberosum *(potato) and *Nicotianna tabacum *(tobacco) and including additional host species of *Solanum *and *Nicotiana*.

A global phylogeographical framework is critical to unravel the answers on large scale-hypotheses on the origins and evolution of *R. solani *AG-3 as a pathogen of domesticated crops within the Solanaceae. A sampling initiative towards the understanding of the phylogeography of populations from potato and tobacco should include the main potato and tobacco growing areas in the world, besides the centers of origin in the Andes mountain valleys of Bolivia, Peru, Columbia and Ecuador, in South America (for hosts on both *Nicotiana *and *Solanum *genera) [52]. It also should include Africa and Australia for the *Nicotiana*, which is part of a truly widespread tribe within the Solanaceae with a Gondwanan distribution (the southern supercontinent, which included most of the landmasses in today's southern hemisphere) [52].

## Conclusion

The two sister populations of the basidiomycete fungus *R. solani *AG-3 from potato and tobacco represent two distinct and historically divergent lineages that have probably evolved within the range of their particular related Solanaceae hosts as sympatric species.

## Methods

### Isolates and populations

**Figure 6:**
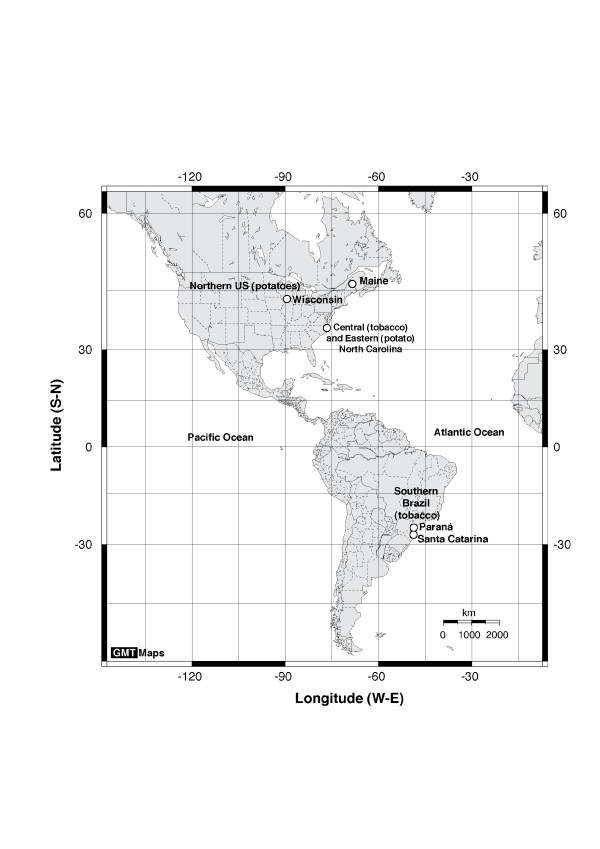
Geographic origin of potato and tobacco isolates of *Rhizoctonia solani *AG-3 collected from Paraná and Santa Catarina States in Brazil and from Maine, North Carolina and Wisconsin in the US.

Isolates from four randomly selected population samples of *R. solani *AG-3 were analyzed: eight potato isolates from Eastern NC and five from the Northern US; and six tobacco isolates from central NC and nine tobacco isolates from southern Brazil (Table 5, Figure 6). These population samples of *R. solani *AG-3 from potato and tobacco constituted subsamples of a larger hierarchical sample formerly studied by Ceresini et al [11,12,21].

**Table 5:** Description and origin of pP42F and pP89 haplotypes of *Rhizoctonia solani *AG-3 from potato and tobacco

Isolate	Origin (region and State)	Loci					
		
		pP42F			pP89		
		
		Haplotypes designation for phylogenetic analysis	Haplotype designation for coalescent analysis*	Sequence code at GenBank^® ^NCBI	Haplotypes designation for phylogenetic analysis	Haplotype designation for coalescent analysis*	Sequence code at GenBan^® ^NCBI
**Potato tubers**	**Northern US**						
P006	Maine	P0061	A	AY458145.1	P0061	A	AY458184.1
		P0062	A	AY458146.1	P0062	L	AY458185.1
P034	Wisconsin	P0341 a/b	G	AY458147.1	P0341	L	AY458186.1
		-	-	-	P0342	L	AY458187.1
P047	Maine	P0471	A	AY458148.1	P0471	K	AY458188.1
		P0472	F	AY458149.1	P0472	G	AY458189.1
P083	Wisconsin	P0831	G	AY458150.1	P08310	L	AY458190.1
		P0832	J	AY458151.1	P08311	L	AY458191.1
P084	Wisconsin	P0841	D	AY458152.1	P0841	H	AY458192.1
		P0842	J	AY458153.1	P0842	B	AY458193.1
**Potato stems**	**Eastern NC**						
P102	Washington	P1021	A	AY458154.1	P1021	I	AY458194.1
		P1022	K	AY458155.1	P1022	C	AY458195.1
P137	Tyrrell	P1371	A	AY458156.1	P1371	L	AY458196.1
		P1372	D	AY458157.1	P1372	B	AY458197.1
P151	Hyde	P1511	K	AY458158.1	P1511	B	AY458198.1
		P1512	H	AY458159.1	P1512	L	AY458199.1
P152	Hyde	P1521	J	AY458160.1	P1522	L	AY458200.1
		P1522	K	AY458161.1	P1523	B	AY458201.1
P170	Hyde	P1701 a/b	D	AY458162.1	P1701 a/b	L	AY458202.1
P198	Camdem	P1981	I	AY458163.1	P1984	L	AY458203.1
		P1982	A	AY458164.1	P1986	D	AY458204.1
P216	Camdem	P2161	J	AY458165.1	P2161	L	AY458205.1
		P2162	E	AY458166.1	P2162	L	AY458206.1
P217	Camdem	P2171	J	AY458167.1	P2171	L	AY458207.1
		P2172	E	AY458168.1	P2172	J	AY458208.1
**Tobacco leaves**	**Central NC**						
T025	Lenoir	T0251 a/b	B	AY458169.1	T0251 a/b	E	AY458209.1
T033	Duplin	T0331 a/b	B	AY458170.1	T0331 a/b	E	AY458210.1
T084	Edgecombe	T0841 a/b	B	AY458171.1	T0841 a/b	E	AY458211.1
T113	Edgecombe	T1131 a/b	B	AY458172.1	T1131 a/b	E	AY458212.1
T149	Caswell	T1491 a/b	B	AY458173.1	T1491 a/b	E	AY458213.1
T168	Caswell	T1681 a/b	B	AY458174.1	T1681 a/b	E	AY458214.1
	**Southern Brazil**						
TBR17	Parana and Santa Catarina States	TBR171 a/b	B	AY458175.1	TBR171 a/b	E	AY458215.1
TBR19		TBR191 a/b	B	AY458176.1	TBR191 a/b	E	AY458216.1
TBR20		TBR201 a/b	B	AY458177.1	TBR201 a/b	E	AY458217.1
TBR21		TBR211 a/b	B	AY458178.1	TBR211 a/b	E	AY458218.1
TBR22		TBR221 a/b	B	AY458179.1	TBR221 a/b	E	AY458219.1
TBR23		TBR231 a/b	B	AY458180.1	TBR231 a/b	E	AY458220.1
TBR24		TBR241 a/b	C	AY458181.1	TBR241 a/b	F	AY458221.1
TBR27		TBR271 a/b	B	AY458182.1	TBR271 a/b	E	AY458222.1
TBR28		TBR281 a/b	B	AY458183.1	TBR281 a/b	E	AY458223.1

### DNA technique for genotyping

We conducted sequence analysis of two PCR amplified nuclear DNA fragments: pP42F, with 401 bp, and pP89, with 1090 bp (Table 6). By BLASTX 2.2.3 [53] analysis with DNA sequences deposited at GenBank^®^, the pP42F marker was found to have sequence similarity to the nuclear gene *lsc2p *from *Saccharomyces cerevisiae *encoding for beta subunit of succinyl-CoA ligase or to a similar gene from *Neocallimastix frontalis*; the pP89 marker had sequence similarity to the nuclear gene *crm1 *from *Schizosaccharomyces pombe *encoding for chromosome region maintenance protein 1 or exportin 1 (Table 6).

**Table 6:** Primers for PCR amplification and sequencing reactions of two nuclear DNA fragments from *Rhizoctonia solani *AG-3 PT and TB

Loci	Product size	Oligo	Length	Tm (°C)	GC%	Sequence (5' to 3')
pP42F ^a^	401	F119L	19	60.4	57.9	GTTGGATCACGTCGCTCAG
		F518R	20	59.7	55.0	TAGTATGGGATACCCACGCC
pP89^b^	1090	F02	21	62.3	52.4	TTTGAGGAAGAACGCGTACGC
		R10	21	60.7	42.9	TGTCATTGAAAATACGGCCGA
	Internal	L253	22	59.9	50.0	GTGTGTACTTGTTGGGGAGACA
	primers	R790	22	59.7	45.5	GGTCGTGGGCAAATCTTAATAC

^a ^Similar to *lsc2p *gene encoding for beta subunit of succinyl-CoA ligase (synthetase; ATP-forming; a mitochondrial enzyme of the TCA cycle) localized at nuclear chromosome VII from *Saccharomyces cerevisiae*, accession 6321683 deposited at GenBank (BLASTX2.2.3 score of 89.0 and E-value of 9e-^31^) or succinyl-CoA ligase [GDP-forming] beta-chain, hydrogenosomal precursor (succinyl-CoA synthetase, beta chain) from *Neocallimastix frontalis*, accession 9789854 (BLASTX2.2.3 score of 99 and E-value of 3e^-33^)

^b ^Similar to *crm1 *gene, encoding for chromosome region maintenance protein 1 or exportin 1 from *Schizosaccharomyces pombe*, accession 19856107 (BLASTX2.2.3 score of 291 and E-value of e^-101^)

PCR reactions were conduced in a Model 9600 DNA ThermoCycler (Perkin-Elmer) employing standard conditions described previously for *R. solani *[12] and using the Expand High Fidelity PCR System (Roche Molecular Biochemicals), which contains a thermostable, exonuclease-free, N-terminal deletion mutant of *Taq *DNA polymerase and a proofreading polymerase (*Pfu*) exhibiting 3'-exonuclease activity. After purification by ultrafiltration with QIAquick PCR Purification Kit (QIAGEN^®^), the PCR products were sequenced in both directions by the dideoxy chain terminating method using the Big Dye Terminator Cycle Sequencing Ready Reaction Kit (Applied Biosystems, Inc.) and analyzed on an ABI 3700 automatic sequencer (Perkin Elmer Corp., Norwalk, CT). Sequence chromatograms were compiled using Sequencher software (vers. 2.0, GeneCodes Corp.).

To separate different pP42F or pP89 alleles within heterogenous PCR reactions, amplicons were cloned into the PCR2.1-TOPO^® ^vector (Invitrogen, San Diego). Plasmids from selected recombinant One Shot^® ^DH5a™-T1R *Escherichia coli *(Invitrogen, San Diego) were extracted from each sample and purified using QIAprep Spin Miniprep Kit (QIAGEN^®^). Selected primers were used to reamplify and sequence the clones from PCR2.1-TOPO^® ^(Table 6). Sequences were aligned using the software Sequencher (vers. 2.0, GeneCodes Corp.).

### Measures of nucleotide diversity and intragenic recombination

Nucleotide diversity, or the average number of differences per site between two homologous sequences (π), was calculated using the program DNASP [54] according to the equation 10.5 of Nei [55]. Diversities were calculated for the population and separately for the two major clades. The spectrum of polymorphism along each locus was measured and represented as π along a sliding window of 25 positions. The minimum number of recombination events between two adjacent sites (Rm) was calculated according to Hudson and Kaplan [24,25]. Recombination parameters were estimated according to Hudson [25].

### Phylogenetic analysis

The phylogenetic analyses were conducted using maximum likelihood (ML) and Bayesian methods. For ML, the software PAUP* 4.0b10 was used [56]. The Bayesian analysis was conducted using the Monte Carlo Markov Chain method coupled with the Metropolis algorithm (MCMCMC), implemented by the software MrBayes v.2.10 [57].

For both ML and MCMCMC analyses, MODELTEST 3.7 [58] was used to determine the DNA bases substitution model more fit to the data. A hierarchical likelihood ratio test implemented by MODELTEST selected the following models for the markers: **a) pP42F**: K80 (K2P)+G model (number of substitution types = 2; transition/transversion ratio = 3.1683 (kappa = 6.3366); assumed proportion of invariable sites = none; assumed nucleotides frequencies = equal; gamma distribution of rates at variable sites, shape parameter (alpha) = 0.1147; under this evolution model for base substitution, the ML value for the pP42F tree was -lnL = 886.9029); **b) pP89**: K80 (K2P)+G+I model (number of substitution types = 2; transition/transversion ratio = 15.4892 (kappa = 30.9784); assumed proportion of invariable sites = 0.8057; assumed nucleotides frequencies = equal; gamma distribution of rates at variable sites, shape parameter (alpha) = 0.5569; under this evolution model for base substitution, the ML value for the pP89 tree was -lnL = 2271.1220).

The phylogenetic analysis by MCMCMC was performed using, as *a priori *hypothesis, the model of DNA bases substitution described above for the ML analysis. We have searched for 3,000,000 generations using four chains of search, eliminating the first 500 trees, and storing one tree every 500. Posterior probability supporting values (PP) were generated for the branches of the consensus tree considering the rule of a minimum of 50% of the 11002 trees showing a particular partition indicated by the branches.

### Intraspecific evolution of *R. solani *AG-3 inferred by analysis of haplotype networks

For inferring intraspecific evolution, networks of haplotypes sampled from populations of *R. solani *AG-3 were built using the algorithm recommended by Posada & Crandall [26]. This method starts by estimating the maximum number or differences between haplotypes as a result of single substitutions (i.e., those not resulting from multiple substitutions in a single site) with the statistical significance level of 95% [59]. This is called the limit of parsimony, or the limit of parsimony connection. Haplotypes differing by only one change are connected, and then those differing by two, three and so on until all the haplotypes are included in a single network, or until the limit of parsimony connection is reached. This method also allows for the identification of probable recombinants through observations of spatial distribution of the sequence of homoplasies defined by the network [60]. The estimation of the phylogeny from DNA sequences was implemented by the computer program TCS [61].

### Population structure

Between-population analyses were conducted to test for geographic structure in populations of *R. solani *AG-3. Pairwise Φ statistics comparisons (F statistics equivalents [62]) were used to describe the proportion of the total genetic variance at each locus due to differences among populations. The sequences were grouped by sample origin into four populations of *R. solani *AG-3: potato tubers from the Northern US; potato stems from Eastern NC; tobacco leaves from Central NC; and tobacco leaves from Southern Brazil. F_ST _was calculated via analysis of molecular variance (AMOVA) using the software package ARLEQUIN [63,64]. AMOVA estimates variance components considering the number of differences between molecular genotypes [63,65,66]. F_ST _ranges from 0.0, in which all populations appears homogenous, to 1.0, in which all the variation is among populations.

### Nested clade analysis

The nested clade analysis (NCA) on haplotypes of *R. solani *AG-3 from potato was performed with the objective of discriminating between phylogenetic patterns that result from historical events (for example, past fragmentation, expansion, colonization) and from those due to recurrent gene flow [34,35]. This analysis utilizes both a phylogeny and the geographical distribution of haplotypes or haplotype clades. The NCA was completed using the computer program GeoDis v.2.0 [67] considering the haplotype network (phylogeny) obtained by statistical parsimony analysis as described above. This analysis divides the phylogeny into nested clades of *n *steps, where *n *is equal to the number of mutations connecting the haplotypes of a clade. For each clade there is a geographic point around which all the individuals are centered. Two quantitative measures of how within-clade haplotypes are geographically dispersed were used: the clade distance = *D*_*c *_(*X*), which describes the average distance of each haplotype within clade *X *from the geographic center of clade *X*; and the nested clade distance = *D*_*n *_(*X*), which similarly describes the average distance of each haplotype within clade *X *from the geographic center of the next more inclusive clade within which the clade *X *is nested [35]. The clade distance *D*_*c *_(*X*) measures the geographical range of a particular clade *X *(average distance that a haplotype lies from the geographical center of all haplotypes from the same clade). The nested clade distance *D*_*n *_(*X*) measures how a particular clade *X *is geographically distributed relatively to its closest evolutionary sister clades (i.e., clades in the same higher level nesting category). Accordingly, within each nested category and for both types of distances (*D*_*c *_and *D*_*n*_), we calculated and determined the significance of the average interior distance minus the average tip distance [(*Int *- *Tip*)_*c *_and (*Int *- *Tip*)_*n*_]. Contrasts of interior versus tip clades within a nested clade strongly indicate contrasts of older versus younger clades. Haplotypes were assigned to the following populations: Maine (46.51562 N 68.36468 W), Wisconsin (43.07980 N 89.38751 W), and the North Carolina counties of Washington (35.83681 N 76.56470 W), Tyrrell (35.87039 N 76.16513 W), Hyde (35.40617 N 76.15316 W) and Camden (36.33743 N 76.16263 W). Clade distances were calculated using a distance matrix among populations based on geographic coordinates. A test of 1000 permutations of population assignments to haplotypes was used to determine the significance of larger or smaller values of *D*_*c*_, *D*_*n*_, (*Int *- *Tip*)_*c *_and (*Int *- *Tip*)_*n *_than would be expected for null hypothesis of random geographical association of haplotypes [35].

### Coalescent analysis

SNAP Workbench Java program package was used to analyze gene genealogies and population parameters [68]. SNAP Map was used to collapse sequences into haplotypes, removing indels and infinite sites violations among the mutations [69]. Coalescent methods make strict assumptions, such as neutrality and the lack of recombination (identified based on the detection of homoplasious sites), which must be verified previously. To test for departures from neutrality, Tajima's D [70] and Fu and Li's D and F* [59,71] test statistics were calculated using the statistical tests of neutrality of mutations against excess of recent mutations (rare alleles), written by Yun-Xin Fu [72]. We hypothesized that any deviation from neutrality was due to change in effective population size. This hypothesis was tested using the population growth estimates inferred by the program Lamarc 2.0 [28,29]. The Lamarc algorithm permits intragenic recombination and therefore allowed us to use all polymorphic sites to assess this population parameter. To estimate the population growth parameter, we used 10 initial chains with 2,000 genealogies sampled and 2 final chains with 10,000 genealogies sampled. The population growth rates were inferred using Bayesian analyses, assessing the 95% credibility intervals from the Bayesian search.

**Figure 7:**
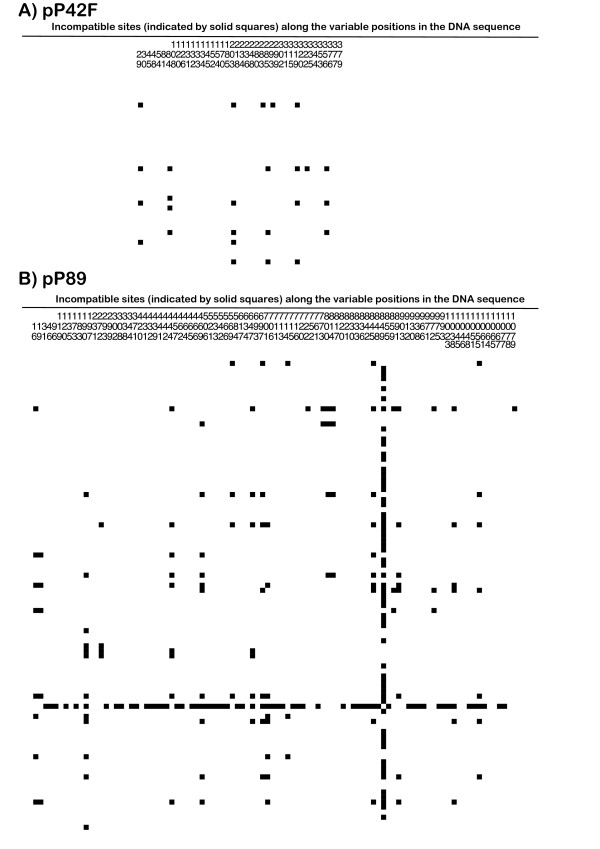
**Site compatibility matrices for *Rhizoctonia solani *AG-3 homoplasious haplotypes in the pP42F (A) and pP89 (B) loci**. The numbers along the top of the matrix indicate variable positions in the DNA sequences alignment of the data. Incompatible sites are indicated by solid squares; all other sites in the matrix are compatible.

To generate compatible sequences alignments for coalescent analyses, the conflicting sites in each alignment were manually removed [46]. Conflicting sites showing homoplasy were initially identified among variable sites using the compatibility methods SNAP Clade and SNAP Matrix (Figure 7-A and 7B). Nine incompatible sites were removed from the pP42F dataset, resulting in 33 polymorphic sites after manipulation of the alignments; from a total of 95 polymorphic sites, 20 incompatible ones were removed from the pP89 alignment dataset.

Based on the evidence for no geographic population structure, we pooled data for the two tobacco populations of *R. solani *AG-3 (US + Brazil) for both loci. Data for the potato populations (Northern US + Eastern NC) were also pooled for the pP42F marker, but kept separate for the pP89. Coalescent parameters were estimated for each locus separately, due to the lack of information on how the alleles at each locus are linked in the haploid phase of the fungus. To determine the order of coalescent events for haplotypes in time it is necessary to determine the amount of migration that occurred between populations. Haplotypes from populations linked by migration are postulated to coalesce before haplotypes from unlinked populations [73]. Using the program Migrate [74], incorporated in the SNAP Workbench program package, migration matrices were constructed for each locus, indicating the number of migrants exchanged between populations. These backward migration matrices were used for ancestral inference in Genetree version 9.0 [74,75], also incorporated in the SNAP Workbench. Subsequently, we reconstructed the genealogy with the highest root probability, the ages of mutation and the TMRCA (time for the most recent common ancestor) of the samples using 100,000 coalescent simulations with five runs with distinct starting random number seeds, considering population subdivision and distinct population sizes. The program allows for the estimation of the ancestral history of each haplotype. It also shows the distribution of mutations on a coalescent scale, allowing for comparison of the divergence of haplotypes between and within each population.

See Additional file 1.xls and Additional file 2.xls for the original data used to perform the analyses from this study.

## Authors' contributions

PCC, TYJ, RJV, HDS, and MAC designed the study. PCC was involved in the sampling and carried out the molecular work. PCC and TYJ analyzed the data and drafted the manuscript. TYJ, RJV, HDS, and MAC helped draft the manuscript. All authors read and approved the final manuscript.

## Supplementary Material

Additional file 1 **Characterization of haplotypes of *Rhizoctonia solani *AG-3 from potato and tobacco according to polymorphic sites detected in pP42F DNA sequences**. ^a ^Conflicting sites showing homoplasy are presented in red; these incompatible sites, among the polymorphic ones, were identified using the compatibility methods SNAP Clade and SNAP Matrix (Figure 7-A). Site types: t = transitions; v = transversions. Character type: i = phylogenetically informative; - = uninformative sites. Click here for file

Additional file 2 **Characterization of haplotypes of *Rhizoctonia solani *AG-3 from potato and tobacco according to polymorphic sites detected in pP89 DNA sequences**. a) Conflicting sites showing homoplasy are presented in red; these incompatible sites, among the polymorphic ones, were identified using the compatibility methods SNAP Clade and SNAP Matrix (Figure 7-B). Site types: t = transitions; v = transversions. Character type: i = phylogenetically informative; - = uninformative sites. Click here for file
